# Synthesis, crystal structure and Hirshfeld surface analysis of a coordination compound of cadmium nitrate with 2-amino­benzoxazole

**DOI:** 10.1107/S2056989025004049

**Published:** 2025-05-09

**Authors:** Surayyo Razzoqova, Yodgor Ruzimov, Akobir Toshov, Batirbay Torambetov, Aziz Ibragimov, Jamshid Ashurov, Shakhnoza Kadirova

**Affiliations:** a National University of Uzbekistan named after Mirzo Ulugbek 4 University St Tashkent 100174 Uzbekistan; b Khorezm Mamun branch of Uzbekistan Academy of Sciences, 1, Markaz St., Khiva, 220900, Uzbekistan; c Physical and Material Chemistry Division CSIR-National Chemical Laboratory,Pune 411008 India; d Institute of General and Inorganic Chemistry, Academy of Sciences of Uzbekistan, M. Ulugbek Str 77a, Tashkent 100170, Uzbekistan; e Institute of Bioorganic Chemistry, Academy of Sciences of Uzbekistan, M. Ulugbek, St, 83, Tashkent, 100125, Uzbekistan; Vienna University of Technology, Austria

**Keywords:** crystal structure, mol­ecular structure, cadmium complex, 2-amino­benzoxazole, octa­hedral coordination

## Abstract

A coordination complex of cadmium nitrate [Cd(NO_3_)_2_] with 2-amino­benzaxole (2AB; C_7_H_6_N_2_O), namely, tetra­kis­(2-amino­benzoxazole-κ*N*^1^)bis­(nitrato-κ*O*)cadmium(II), [Cd(NO_3_)_2_(2AB)_4_], has been synthesized from ethanol solutions of Cd(NO_3_)_2_·H_2_O and 2AB. The asymmetric unit comprises half a mol­ecule of [Cd(NO_3_)_2_(2AB)_4_], with the Cd^II^ atom positioned on a twofold rotation axis. In the completed mol­ecular complex, four 2AB ligands and two nitrate anions each coordinate monodentately to the Cd^II^ atom, leading to a distorted octa­hedral coordination environment. The crystal structure of [Cd(NO_3_)_2_(2AB)_4_] exhibits several N—H⋯O inter­actions, resulting in the formation of a layered assembly parallel to (001). Hishfeld surface analysis was used to qu­antify the inter­molecular inter­actions.

## Abstract 2

The Cd^II^ atom in the title complex [Cd(NO_3_)_2_(2AB)_4_] (2AB is 2-amino­benzaxole; C_7_H_6_N_2_O) has a distorted octa­hedral coordination environment. In the crystal structure, several N—H⋯O inter­actions lead to the formation of layers parallel to (001).

## 1. Chemical context

Benzoxazole is a heterocyclic aromatic compound consisting of a benzene ring fused to an oxazole ring. It has a strong and unpleasant fishy odour, just like pyridine (Katritzky & Pozharskii, 2000 ▸; Clayden *et al.*, 2001 ▸). Many benzoxazole-based compounds are valued in medicinal and biological research because of their numerous biological activities (Potashman *et al.*, 2007 ▸; Šlachtová. & Brulíková, 2018 ▸; Razzoqova *et al.*, 2022 ▸, 2024 ▸), including anti­microbial (Erol *et al.*, 2022 ▸), anti­tumor (Imaizumi *et al.*, 2020 ▸), anti-inflammatory (Parlapalli & Manda, 2017 ▸), analgesic (Ali *et al.*, 2022 ▸; Sattar *et al.*, 2020 ▸), anti­tubercular (Šlachtová & Brulíková, 2018 ▸), herbicidal (Sangi *et al.*, 2019 ▸), and fungicidal properties (Fan *et al.*, 2022 ▸).
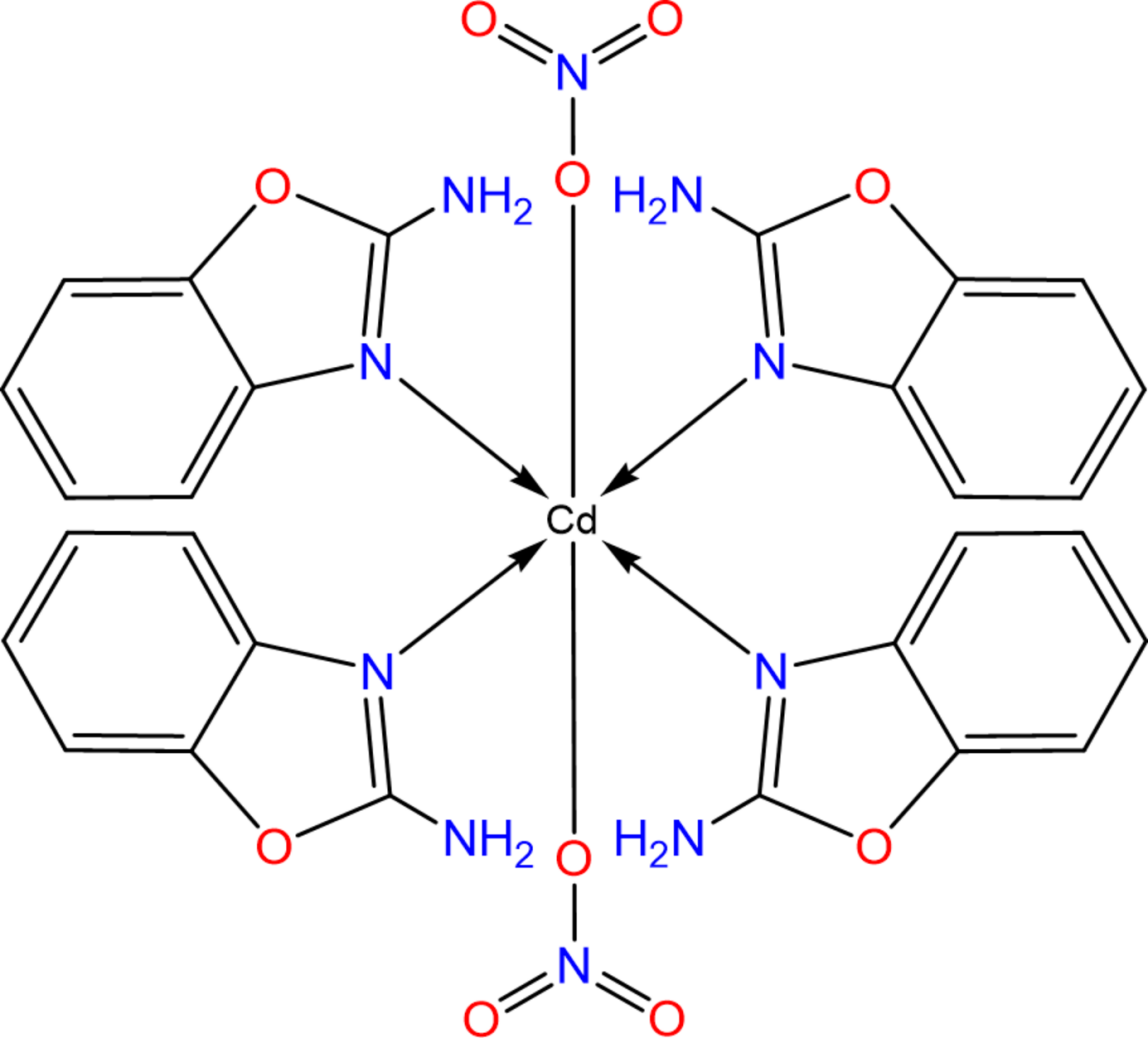
At the same time, 2-amino­benzoxazole (2AB) and its derivatives have potent anti­bacterial and anti­cancer properties (Paramashivappa *et al.*, 2003 ▸; Khajondetchairit *et al.*, 2017 ▸; Ouyang *et al.*, 2012 ▸). One notable derivative of 2AB is 2-amino-5-chloro­benzoxazole, which has demonstrated muscle relaxant effects and is used as an anti­spasmodic and uricosuric agent in therapeutic applications (Lynch, 2004 ▸).

In the context given above, we present here the synthesis, crystal structure determination and Hirshfeld surface analysis of a coordination complex of 2AB with cadmium nitrate, [Cd(NO_3_)_2_(2AB)_4_].

## 2. Structural commentary

**Figure 1:**
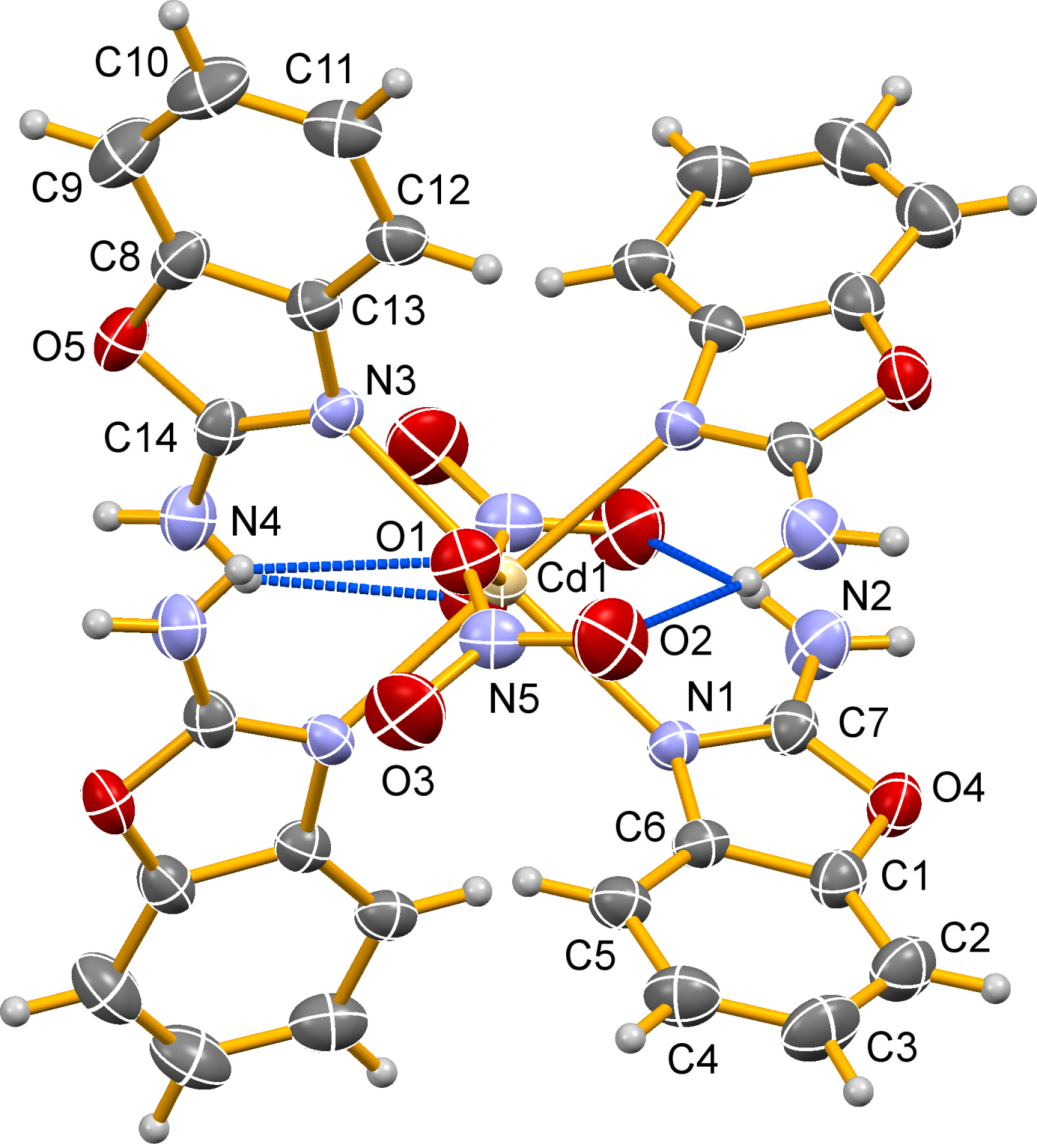
The mol­ecular structure of [Cd(NO_3_)_2_(2AB)_4_] with displacement ellipsoids drawn at the 30% probability level; non-labelled atoms are generated by symmetry code −*x* + 1, *y*, −*z* + 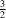. Intra­molecular hydrogen bonds are indicated by dashed blue lines.

In the asymmetric unit of [Cd(NO_3_)_2_(2AB)_4_], which consists of half of a complex molecule, the Cd^II^ atom is positioned on a twofold rotation axis (multiplicity 4, Wyckoff letter *e*). In the completed mol­ecule, the Cd^II^ atom coordinates by four 2AB ligands and two nitrate anions, resulting in a distorted octa­hedral N_4_O_2_ coordination set (Fig. 1 ▸). The four 2AB ligands occupy the equatorial positions and are coordinated monodentately through their aromatic nitro­gen donor atoms with Cd—N bond lengths of 2.314 (3) and 2.325 (3) Å. The two axially positioned nitrato ligands are also coordinated in a monodentate fashion with a relatively long Cd—O bond length of 2.418 (3) Å. The dihedral angle formed between the two opposite 2-amino­benzaxazole ligands (labelled in Fig. 1 ▸) is 84.85 (17)°. The mol­ecular conformation is stabilized by intra­molecular N—H⋯O hydrogen-bonding inter­actions involving the coordinated oxygen atom O1 and the non-coordinated oxygen atom O2 (entries #1 and #3 in Table 1 ▸).

## 3. Supra­molecular features

**Figure 2:**
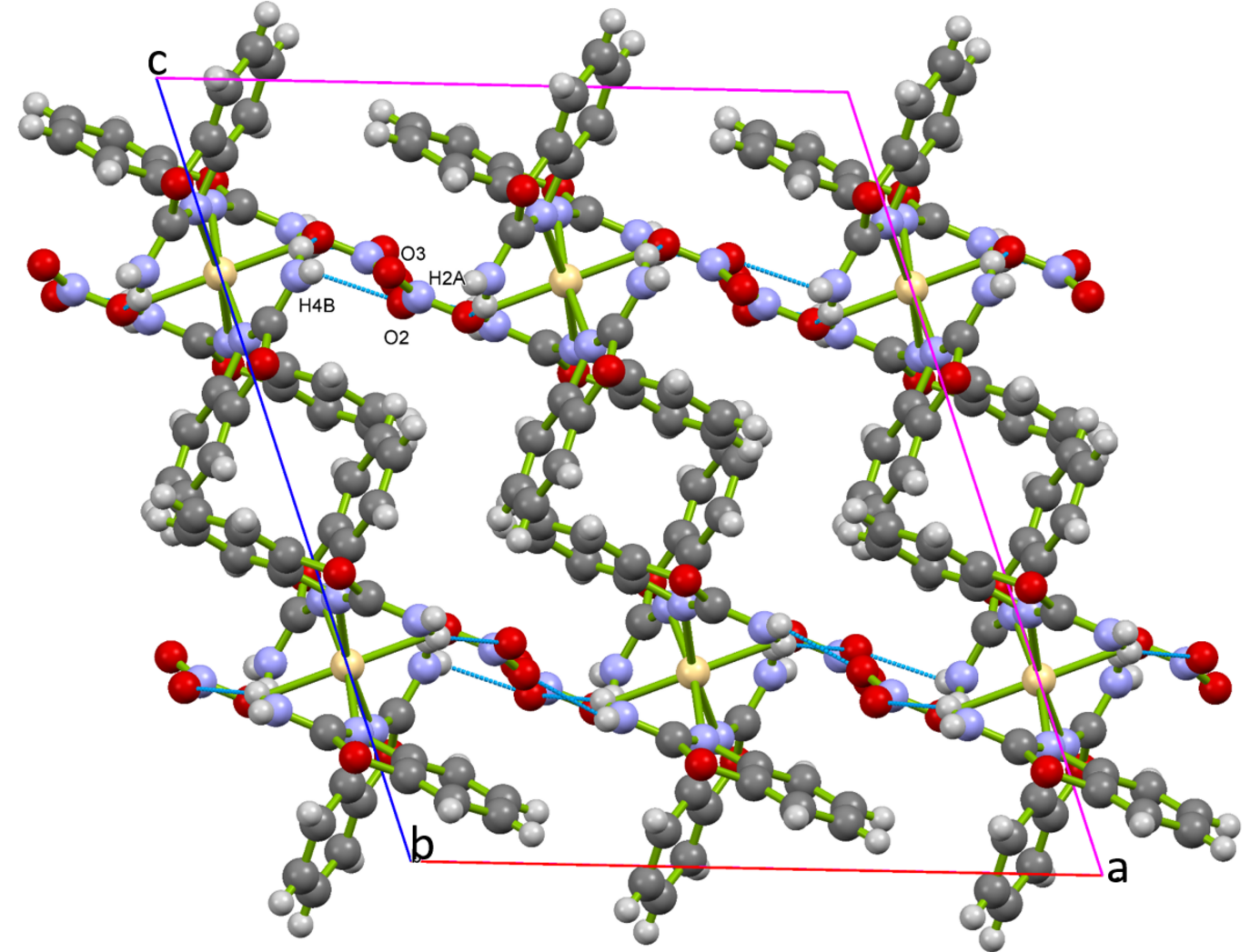
Visualization of the mol­ecular packing in [Cd(NO_3_)_2_(2AB)_4_] in a view along [010]. Inter­molecular N—H⋯O inter­actions are shows as light-blue dashed lines.

In the crystal structure of [Cd(NO_3_)_2_(2AB)_4_], inter­molecular N—H⋯O hydrogen bonds involving the non-coordinated O atoms O2 and O3 (entries #2 and #4 in Table 1 ▸) lead to the formation of sheets extending parallel to (001), as shown in Fig. 2 ▸.

## 4. Hirshfeld Surface Analysis

**Figure 3:**
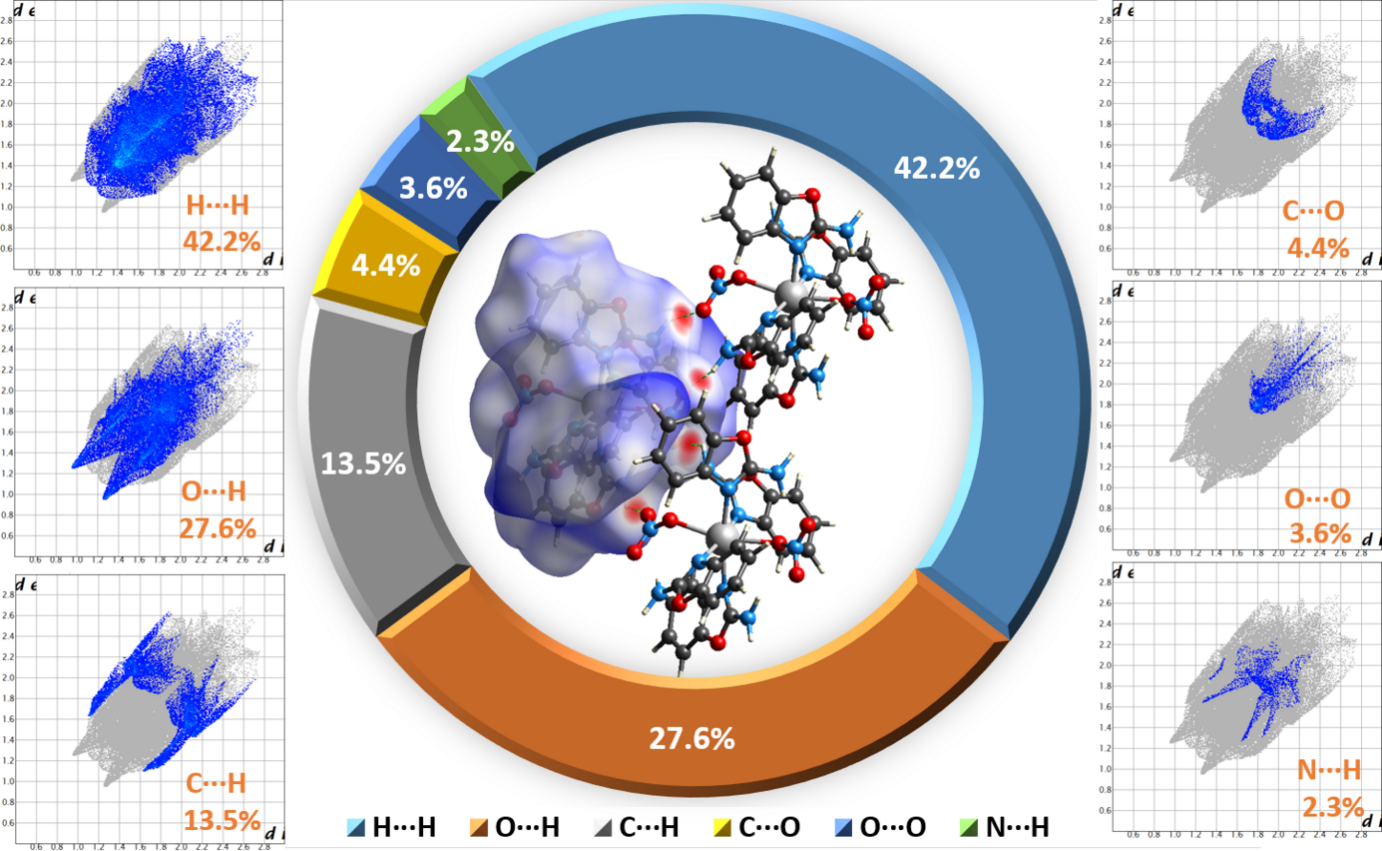
View of HS and two-dimensional fingerprint plots of [Cd(NO_3_)_2_(2AB)_4_].

Hirshfeld surface (HS) analysis (Spackman & Jayatilaka, 2009 ▸) was performed and two-dimensional fingerprint plots (Spackman & McKinnon, 2002 ▸) were generated using *Crystal­Explorer* (Spackman *et al.*, 2021 ▸) to qu­antify the inter­molecular inter­actions. HS and fingerprint plot analysis conducted for [Cd(NO_3_)_2_(2AB)_4_] are graphically displayed in Fig. 3 ▸. The red spots on the HS area of [Cd(NO_3_)_2_(2AB)_4_] confirm the close inter­molecular N—H⋯O contacts (related to entries #2 and #4 in Table 1 ▸) between adjacent mol­ecules. The two-dimensional fingerprint plots and their relative contributions revealed that H⋯H, O⋯H, C⋯H, C⋯O, O⋯O and N⋯H inter­actions are the main inter­actions to the HS area. Specifically, the fingerprint plots reveal the presence of close N—H⋯O contacts in form of two spikes observed near (*d*_i_ + *d*_e_) ≃ 2.3 Å and C—H contacts as two wings near (*d*_i_ + *d*_e_) ≃ 2. 8 Å (Fig. 3 ▸).

## 5. Database survey

A survey of the Cambridge Structural Database (CSD, Version 5.46, November 2024; Groom *et al.*, 2016 ▸) revealed 17 crystal structures of 2-amino­benzoxazole derivatives. Among these, only two structures involve coordination compounds with zinc (QALXIL; Decken & Gossage, 2005 ▸) and cadmium (DIWPIM; Razzoqova *et al.*, 2023 ▸). In the zinc complex, the central metal atom coordinates two benzoxazolamine ligands through the aromatic nitro­gen atom and two chloro ligands in a distorted tetra­hedral coordination environment. In the crystal structure of DIWPIM, which corresponds to [Cd(2AB)_2_(CH_3_COO)_2_], the Cd^II^ atom coordinates by two 2AB ligands and two acetato ligands in a monodentate and bidentate fashion, respectively, forming a distorted octa­hedral N_2_O_4_ coordination set.

## 6. Synthesis and crystallization

Cd(NO_3_)_2_·H_2_O (0.308 g, 1 mmol) and 2AB (0.268 g, 2 mmol) were dissolved separately in ethanol (5 ml), mixed together and stirred for 2 h. The obtained colourless solution was filtered and left for crystallization. Single crystals of the complex [Cd(NO_3_)_2_(2AB)_4_] suitable for X-ray analysis were obtained by slow evaporation of the solution over a period of 7 d.

## 7. Refinement

Crystal data, data collection and structure refinement details are summarized in Table 2 ▸. Hydrogen atoms were treated in a riding model with *U*_iso_(H) = 1.2*U*_eq_(C,N).

## Supplementary Material

Crystal structure: contains datablock(s) I. DOI: 10.1107/S2056989025004049/wm5756sup1.cif

Structure factors: contains datablock(s) I. DOI: 10.1107/S2056989025004049/wm5756Isup2.hkl

CCDC reference: 2448896

Additional supporting information:  crystallographic information; 3D view; checkCIF report

Additional supporting information:  crystallographic information; 3D view; checkCIF report

## Figures and Tables

**Table 1:** Hydrogen-bond geometry (Å, °)

*D*—H⋯*A*	*D*—H	H⋯*A*	*D*⋯*A*	*D*—H⋯*A*
N4—H4*A*⋯O1^i^	0.86	2.26	2.971 (5)	140
N4—H4*B*⋯O2^ii^	0.86	2.28	2.822 (6)	121
N2—H2*A*⋯O2^i^	0.86	2.11	2.899 (7)	152
N2—H2*B*⋯O3^iii^	0.86	2.33	2.953 (6)	129

Symmetry codes: (i) 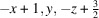; (ii) 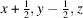; (iii) 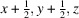.

**Table 2:** Experimental details

Crystal data	
Chemical formula	[Cd(NO_3_)_2_(C_7_H_6_N_2_O)_4_]
*M*_r_	772.97
Crystal system, space group	Monoclinic, *C*2/*c*
Temperature (K)	293
*a*, *b*, *c* (Å)	15.9012 (3), 11.0897 (2), 18.9475 (5)
β (°)	109.182 (3)
*V* (Å^3^)	3155.70 (13)
*Z*	4
Radiation type	Cu *K*α
μ (mm^−1^)	6.19
Crystal size (mm)	0.10 × 0.08 × 0.06

Data collection	
Diffractometer	XtaLAB Synergy, Single source at home/near, HyPix3000
Absorption correction	Multi-scan (*CrysAlis PRO*; Rigaku OD, 2020 ▸)
*T*_min_, *T*_max_	0.016, 1.000
No. of measured, independent and observed [*I* > 2σ(*I*)] reflections	12616, 3014, 2324
*R*_int_	0.084
(sin θ/λ)_max_ (Å^−1^)	0.614

Refinement	
*R*[*F*^2^ > 2σ(*F*^2^)], *wR*(*F*^2^), *S*	0.049, 0.128, 1.01
No. of reflections	3014
No. of parameters	223
H-atom treatment	H-atom parameters constrained
Δρ_max_, Δρ_min_ (e Å^−3^)	0.56, −0.95

Computer programs: *CrysAlis PRO* (Rigaku OD, 2020 ▸), *SHELXT* (Sheldrick, 2015*a* ▸), *SHELXL* (Sheldrick, 2015*b* ▸), *OLEX2* (Dolomanov *et al.*, 2009 ▸), *Mercury* (Macrae *et al.*, 2020 ▸) and *publCIF* (Westrip, 2010 ▸).

## supplementary crystallographic information

### Tetrakis(2-aminobenzoxazole-κN1)bis(nitrato-κO)cadmium(II) Crystal data

**Table:**

[Cd(NO_3_)_2_(C_7_H_6_N_2_O)_4_]	*F*(000) = 1560
*M**_r_* = 772.97	*D*_x_ = 1.627 Mg m^−^^3^
Monoclinic, *C*2/*c*	Cu *K*α radiation, λ = 1.54184 Å
*a* = 15.9012 (3) Å	Cell parameters from 4500 reflections
*b* = 11.0897 (2) Å	θ = 4.9–70.8°
*c* = 18.9475 (5) Å	µ = 6.19 mm^−^^1^
β = 109.182 (3)°	*T* = 293 K
*V* = 3155.70 (13) Å^3^	Block, colourless
*Z* = 4	0.10 × 0.08 × 0.06 mm

### Tetrakis(2-aminobenzoxazole-κN1)bis(nitrato-κO)cadmium(II) Data collection

**Table:**

XtaLAB Synergy, Single source at home/near, HyPix3000 diffractometer	3014 independent reflections
Radiation source: micro-focus sealed X-ray tube, PhotonJet (Cu) X-ray Source	2324 reflections with *I* > 2σ(*I*)
Mirror monochromator	*R*_int_ = 0.084
Detector resolution: 10.0000 pixels mm^-1^	θ_max_ = 71.1°, θ_min_ = 4.9°
ω scans	*h* = −19→19
Absorption correction: multi-scan (CrysAlisPro; Rigaku OD, 2020)	*k* = −13→13
*T*_min_ = 0.016, *T*_max_ = 1.000	*l* = −22→23
12616 measured reflections	

### Tetrakis(2-aminobenzoxazole-κN1)bis(nitrato-κO)cadmium(II) Refinement

**Table:**

Refinement on *F*^2^	Hydrogen site location: inferred from neighbouring sites
Least-squares matrix: full	H-atom parameters constrained
*R*[*F*^2^ > 2σ(*F*^2^)] = 0.049	*w* = 1/[σ^2^(*F*_o_^2^) + (0.0631*P*)^2^] where *P* = (*F*_o_^2^ + 2*F*_c_^2^)/3
*wR*(*F*^2^) = 0.128	(Δ/σ)_max_ < 0.001
*S* = 1.01	Δρ_max_ = 0.56 e Å^−^^3^
3014 reflections	Δρ_min_ = −0.95 e Å^−^^3^
223 parameters	Extinction correction: SHELXL (Sheldrick, 2015b), Fc^*^=kFc[1+0.001xFc^2^λ^3^/sin(2θ)]^-1/4^
0 restraints	Extinction coefficient: 0.00041 (6)

### Tetrakis(2-aminobenzoxazole-κN1)bis(nitrato-κO)cadmium(II) Special details

**Table:**

Geometry. All esds (except the esd in the dihedral angle between two l.s. planes) are estimated using the full covariance matrix. The cell esds are taken into account individually in the estimation of esds in distances, angles and torsion angles; correlations between esds in cell parameters are only used when they are defined by crystal symmetry. An approximate (isotropic) treatment of cell esds is used for estimating esds involving l.s. planes.

### Tetrakis(2-aminobenzoxazole-κN1)bis(nitrato-κO)cadmium(II) Fractional atomic coordinates and isotropic or equivalent isotropic displacement parameters (Å^2^)

**Table:**

	*x*	*y*	*z*	*U*_iso_*/*U*_eq_	
Cd1	0.500000	0.24505 (3)	0.750000	0.04211 (19)	
O5	0.5198 (2)	−0.0974 (3)	0.6354 (2)	0.0633 (9)	
O4	0.5320 (2)	0.5949 (3)	0.8680 (2)	0.0650 (9)	
O1	0.3406 (2)	0.2156 (3)	0.7009 (2)	0.0639 (9)	
N1	0.5181 (2)	0.3989 (3)	0.8371 (2)	0.0461 (8)	
N3	0.5049 (2)	0.0945 (3)	0.6668 (2)	0.0470 (8)	
N5	0.2779 (2)	0.2434 (4)	0.7254 (3)	0.0572 (11)	
N4	0.6112 (2)	−0.0366 (4)	0.7492 (2)	0.0625 (11)	
H4A	0.629684	0.016170	0.784177	0.075*	
H4B	0.634011	−0.107721	0.755194	0.075*	
O2	0.2525 (3)	0.3475 (4)	0.7173 (3)	0.1036 (16)	
N2	0.6252 (3)	0.5215 (4)	0.8094 (3)	0.0752 (13)	
H2A	0.645052	0.464034	0.788780	0.090*	
H2B	0.647782	0.592541	0.812309	0.090*	
O3	0.2472 (3)	0.1686 (5)	0.7563 (3)	0.1067 (16)	
C5	0.3927 (3)	0.3589 (5)	0.8882 (3)	0.0590 (12)	
H5	0.386358	0.277102	0.876857	0.071*	
C6	0.4556 (3)	0.4272 (4)	0.8722 (2)	0.0483 (10)	
C13	0.4434 (3)	0.0736 (4)	0.5961 (3)	0.0487 (10)	
C7	0.5601 (3)	0.5007 (4)	0.8366 (3)	0.0527 (11)	
C12	0.3822 (3)	0.1470 (5)	0.5465 (3)	0.0560 (11)	
H12	0.374376	0.226672	0.558271	0.067*	
C1	0.4640 (3)	0.5490 (4)	0.8903 (3)	0.0591 (12)	
C2	0.4121 (4)	0.6093 (5)	0.9230 (3)	0.0768 (16)	
H2	0.418750	0.691342	0.933603	0.092*	
C11	0.3324 (3)	0.0975 (6)	0.4779 (3)	0.0754 (16)	
H11	0.291093	0.145209	0.442908	0.091*	
C14	0.5477 (3)	−0.0084 (4)	0.6863 (3)	0.0501 (11)	
C8	0.4530 (3)	−0.0457 (4)	0.5772 (3)	0.0595 (12)	
C4	0.3384 (4)	0.4174 (6)	0.9221 (3)	0.0793 (17)	
H4	0.294517	0.373897	0.933595	0.095*	
C3	0.3487 (4)	0.5396 (7)	0.9391 (4)	0.087 (2)	
H3	0.311723	0.575692	0.962182	0.105*	
C10	0.3436 (4)	−0.0227 (7)	0.4611 (4)	0.086 (2)	
H10	0.308806	−0.053870	0.415260	0.103*	
C9	0.4051 (4)	−0.0967 (6)	0.5107 (3)	0.0831 (18)	
H9	0.413245	−0.176568	0.499456	0.100*	

### Tetrakis(2-aminobenzoxazole-κN1)bis(nitrato-κO)cadmium(II) Atomic displacement parameters (Å^2^)

**Table:**

	*U*^11^	*U*^22^	*U*^33^	*U*^12^	*U*^13^	*U*^23^
Cd1	0.0425 (2)	0.0410 (3)	0.0463 (3)	0.000	0.01946 (17)	0.000
O5	0.068 (2)	0.0510 (17)	0.074 (2)	0.0032 (16)	0.0276 (18)	−0.0150 (17)
O4	0.073 (2)	0.0507 (18)	0.074 (2)	−0.0063 (16)	0.0271 (18)	−0.0097 (17)
O1	0.0380 (16)	0.081 (2)	0.075 (2)	0.0023 (15)	0.0215 (16)	−0.0023 (18)
N1	0.0500 (19)	0.0458 (18)	0.046 (2)	−0.0011 (16)	0.0208 (16)	−0.0034 (16)
N3	0.0495 (18)	0.0491 (19)	0.047 (2)	0.0014 (16)	0.0214 (16)	−0.0052 (16)
N5	0.0346 (17)	0.077 (3)	0.060 (2)	0.0003 (18)	0.0162 (17)	0.000 (2)
N4	0.059 (2)	0.053 (2)	0.075 (3)	0.0158 (18)	0.022 (2)	0.002 (2)
O2	0.100 (3)	0.096 (3)	0.126 (4)	0.050 (3)	0.053 (3)	0.018 (3)
N2	0.068 (3)	0.073 (3)	0.094 (4)	−0.025 (2)	0.039 (3)	−0.017 (3)
O3	0.107 (3)	0.113 (4)	0.126 (4)	−0.044 (3)	0.074 (3)	−0.008 (3)
C5	0.063 (3)	0.066 (3)	0.053 (3)	0.001 (2)	0.026 (2)	0.002 (2)
C6	0.049 (2)	0.056 (2)	0.041 (2)	0.006 (2)	0.0149 (18)	−0.0022 (19)
C13	0.046 (2)	0.054 (2)	0.049 (3)	−0.0074 (19)	0.0186 (19)	−0.003 (2)
C7	0.053 (2)	0.052 (2)	0.051 (3)	−0.007 (2)	0.014 (2)	−0.007 (2)
C12	0.058 (3)	0.067 (3)	0.049 (3)	−0.005 (2)	0.025 (2)	−0.001 (2)
C1	0.062 (3)	0.055 (3)	0.058 (3)	0.008 (2)	0.018 (2)	−0.001 (2)
C2	0.084 (4)	0.073 (4)	0.077 (4)	0.017 (3)	0.032 (3)	−0.009 (3)
C11	0.061 (3)	0.106 (5)	0.060 (3)	−0.008 (3)	0.019 (3)	0.003 (3)
C14	0.046 (2)	0.048 (2)	0.062 (3)	0.0033 (18)	0.025 (2)	−0.004 (2)
C8	0.062 (3)	0.058 (3)	0.067 (3)	−0.007 (2)	0.031 (2)	−0.015 (2)
C4	0.075 (3)	0.100 (5)	0.071 (4)	−0.005 (3)	0.035 (3)	0.000 (3)
C3	0.083 (4)	0.103 (5)	0.084 (4)	0.027 (4)	0.038 (3)	−0.017 (4)
C10	0.073 (4)	0.114 (5)	0.067 (4)	−0.019 (4)	0.019 (3)	−0.033 (4)
C9	0.079 (4)	0.084 (4)	0.086 (5)	−0.016 (3)	0.027 (3)	−0.035 (3)

### Tetrakis(2-aminobenzoxazole-κN1)bis(nitrato-κO)cadmium(II) Geometric parameters (Å, º)

**Table:**

Cd1—O1^i^	2.418 (3)	N2—C7	1.319 (6)
Cd1—O1	2.418 (3)	C5—H5	0.9300
Cd1—N1^i^	2.325 (3)	C5—C6	1.365 (6)
Cd1—N1	2.325 (3)	C5—C4	1.396 (7)
Cd1—N3	2.314 (3)	C6—C1	1.390 (6)
Cd1—N3^i^	2.314 (3)	C13—C12	1.375 (6)
O5—C14	1.350 (5)	C13—C8	1.392 (6)
O5—C8	1.380 (6)	C12—H12	0.9300
O4—C7	1.349 (5)	C12—C11	1.392 (7)
O4—C1	1.381 (6)	C1—C2	1.358 (7)
O1—N5	1.269 (5)	C2—H2	0.9300
N1—C6	1.401 (6)	C2—C3	1.383 (8)
N1—C7	1.314 (5)	C11—H11	0.9300
N3—C13	1.395 (6)	C11—C10	1.396 (9)
N3—C14	1.318 (5)	C8—C9	1.363 (7)
N5—O2	1.217 (5)	C4—H4	0.9300
N5—O3	1.206 (6)	C4—C3	1.391 (8)
N4—H4A	0.8600	C3—H3	0.9300
N4—H4B	0.8600	C10—H10	0.9300
N4—C14	1.322 (6)	C10—C9	1.381 (9)
N2—H2A	0.8600	C9—H9	0.9300
N2—H2B	0.8600		
			
O1—Cd1—O1^i^	164.46 (17)	C1—C6—N1	108.1 (4)
N1—Cd1—O1^i^	87.51 (12)	C12—C13—N3	132.4 (4)
N1—Cd1—O1	104.00 (12)	C12—C13—C8	119.9 (4)
N1^i^—Cd1—O1^i^	104.00 (12)	C8—C13—N3	107.7 (4)
N1^i^—Cd1—O1	87.51 (12)	N1—C7—O4	114.8 (4)
N1^i^—Cd1—N1	85.58 (18)	N1—C7—N2	128.3 (4)
N3—Cd1—O1	84.62 (12)	N2—C7—O4	116.9 (4)
N3—Cd1—O1^i^	84.18 (13)	C13—C12—H12	121.2
N3^i^—Cd1—O1	84.18 (13)	C13—C12—C11	117.6 (5)
N3^i^—Cd1—O1^i^	84.63 (12)	C11—C12—H12	121.2
N3—Cd1—N1	171.33 (11)	O4—C1—C6	107.7 (4)
N3^i^—Cd1—N1	94.01 (14)	C2—C1—O4	127.7 (5)
N3^i^—Cd1—N1^i^	171.33 (11)	C2—C1—C6	124.6 (5)
N3—Cd1—N1^i^	94.01 (14)	C1—C2—H2	122.5
N3^i^—Cd1—N3	87.70 (18)	C1—C2—C3	115.0 (6)
C14—O5—C8	104.7 (4)	C3—C2—H2	122.5
C7—O4—C1	104.9 (4)	C12—C11—H11	119.6
N5—O1—Cd1	132.6 (3)	C12—C11—C10	120.8 (6)
C6—N1—Cd1	124.1 (3)	C10—C11—H11	119.6
C7—N1—Cd1	124.8 (3)	N3—C14—O5	114.5 (4)
C7—N1—C6	104.6 (4)	N3—C14—N4	129.0 (4)
C13—N3—Cd1	127.1 (3)	N4—C14—O5	116.5 (4)
C14—N3—Cd1	124.3 (3)	O5—C8—C13	108.1 (4)
C14—N3—C13	105.1 (4)	C9—C8—O5	128.0 (5)
O2—N5—O1	116.9 (5)	C9—C8—C13	123.9 (5)
O3—N5—O1	120.2 (5)	C5—C4—H4	119.5
O3—N5—O2	122.9 (5)	C3—C4—C5	121.0 (6)
H4A—N4—H4B	120.0	C3—C4—H4	119.5
C14—N4—H4A	120.0	C2—C3—C4	122.2 (6)
C14—N4—H4B	120.0	C2—C3—H3	118.9
H2A—N2—H2B	120.0	C4—C3—H3	118.9
C7—N2—H2A	120.0	C11—C10—H10	119.1
C7—N2—H2B	120.0	C9—C10—C11	121.8 (5)
C6—C5—H5	121.5	C9—C10—H10	119.1
C6—C5—C4	117.0 (5)	C8—C9—C10	116.0 (6)
C4—C5—H5	121.5	C8—C9—H9	122.0
C5—C6—N1	131.8 (4)	C10—C9—H9	122.0
C5—C6—C1	120.2 (5)		
			
Cd1—O1—N5—O2	79.2 (6)	C13—N3—C14—O5	−0.8 (5)
Cd1—O1—N5—O3	−99.7 (5)	C13—N3—C14—N4	−178.2 (5)
Cd1—N1—C6—C5	26.7 (6)	C13—C12—C11—C10	0.9 (8)
Cd1—N1—C6—C1	−151.9 (3)	C13—C8—C9—C10	−0.6 (9)
Cd1—N1—C7—O4	152.3 (3)	C7—O4—C1—C6	1.0 (5)
Cd1—N1—C7—N2	−27.5 (7)	C7—O4—C1—C2	−177.8 (5)
Cd1—N3—C13—C12	22.8 (7)	C7—N1—C6—C5	179.3 (5)
Cd1—N3—C13—C8	−158.9 (3)	C7—N1—C6—C1	0.7 (5)
Cd1—N3—C14—O5	159.4 (3)	C12—C13—C8—O5	178.4 (4)
Cd1—N3—C14—N4	−18.0 (7)	C12—C13—C8—C9	0.5 (8)
O5—C8—C9—C10	−178.0 (5)	C12—C11—C10—C9	−1.1 (9)
O4—C1—C2—C3	179.8 (5)	C1—O4—C7—N1	−0.6 (5)
N1—C6—C1—O4	−1.1 (5)	C1—O4—C7—N2	179.2 (4)
N1—C6—C1—C2	177.8 (5)	C1—C2—C3—C4	−0.9 (9)
N3—C13—C12—C11	177.5 (5)	C11—C10—C9—C8	0.8 (9)
N3—C13—C8—O5	−0.2 (5)	C14—O5—C8—C13	−0.2 (5)
N3—C13—C8—C9	−178.1 (5)	C14—O5—C8—C9	177.5 (5)
C5—C6—C1—O4	−179.9 (4)	C14—N3—C13—C12	−177.7 (5)
C5—C6—C1—C2	−1.0 (8)	C14—N3—C13—C8	0.6 (5)
C5—C4—C3—C2	0.5 (10)	C8—O5—C14—N3	0.6 (5)
C6—N1—C7—O4	−0.1 (5)	C8—O5—C14—N4	178.4 (4)
C6—N1—C7—N2	−179.8 (5)	C8—C13—C12—C11	−0.7 (7)
C6—C5—C4—C3	−0.3 (8)	C4—C5—C6—N1	−177.9 (5)
C6—C1—C2—C3	1.1 (8)	C4—C5—C6—C1	0.5 (7)

Symmetry code: (i) −*x*+1, *y*, −*z*+3/2.

### Tetrakis(2-aminobenzoxazole-κN1)bis(nitrato-κO)cadmium(II) Hydrogen-bond geometry (Å, º)

**Table:**

*D*—H···*A*	*D*—H	H···*A*	*D*···*A*	*D*—H···*A*
N4—H4*A*···O1^i^	0.86	2.26	2.971 (5)	140
N4—H4*B*···O2^ii^	0.86	2.28	2.822 (6)	121
N2—H2*A*···O2^i^	0.86	2.11	2.899 (7)	152
N2—H2*B*···O3^iii^	0.86	2.33	2.953 (6)	129

Symmetry codes: (i) −*x*+1, *y*, −*z*+3/2; (ii) *x*+1/2, *y*−1/2, *z*; (iii) *x*+1/2, *y*+1/2, *z*.
